# How arginine derivatives alter the stability of lipid membranes: dissecting the roles of side chains, backbone and termini

**DOI:** 10.1007/s00249-021-01503-x

**Published:** 2021-03-04

**Authors:** Sarah F. Verbeek, Neha Awasthi, Nikolas K. Teiwes, Ingo Mey, Jochen S. Hub, Andreas Janshoff

**Affiliations:** 1 Department of Chemistry, Institute of Physical Chemistry, Georg-August-Universität Göttingen, 37077 Göttingen, Germany; 2 Institute of Microbiology and Genetics, Georg-August-Universität Göttingen, 37077 Göttingen, Germany; 3 Theoretical Physics and Center for Biophyics, Saarland University, 66123 Saarbrücken, Germany

**Keywords:** Arginine, CPP, MD-simulation, Breakthrough force

## Abstract

Arginine (R)-rich peptides constitute the most relevant class of cell-penetrating peptides and other membrane-active peptides that can translocate across the cell membrane or generate defects in lipid bilayers such as water-filled pores. The mode of action of R-rich peptides remains a topic of controversy, mainly because a quantitative and energetic understanding of arginine effects on membrane stability is lacking. Here, we explore the ability of several oligo-arginines R$_n$ and of an arginine side chain mimic R$_\mathrm {Side}$ to induce pore formation in lipid bilayers employing MD simulations, free-energy calculations, breakthrough force spectroscopy and leakage assays. Our experiments reveal that R$_\mathrm {Side}$ but not R$_n$ reduces the line tension of a membrane with anionic lipids. While R$_n$ peptides form a layer on top of a partly negatively charged lipid bilayer, R$_\mathrm {Side}$ leads to its disintegration. Complementary, our simulations show R$_\mathrm {Side}$ causes membrane thinning and area per lipid increase beside lowering the pore nucleation free energy. Model polyarginine R$_8$ similarly promoted pore formation in simulations, but without overall bilayer destabilization. We conclude that while the guanidine moiety is intrinsically membrane-disruptive, poly-arginines favor pore formation in negatively charged membranes via a different mechanism. Pore formation by R-rich peptides seems to be counteracted by lipids with PC headgroups. We found that long R$_n$ and R$_\mathrm {Side}$ but not short R$_n$ reduce the free energy of nucleating a pore. In short R$_n$, the substantial effect of the charged termini prevent their membrane activity, rationalizing why only longer $\mathrm {R}_{n}$ are membrane-active.

**Supplementary Information:**

The online version contains supplementary material available at 10.1007/s00249-021-01503-x.

## Introduction

Cell-penetrating peptides (CPPs) are able to cross cell membranes, without being cytotoxic. Therefore, development of novel CPPs for the purpose of drug delivery or for neuroprotective applications have gained enormous interest in recent decades (Gasparini et al. 2015; Guidotti et al. 2017; Gestin et al. 2017; Milletti 2012; Kauffman et al. 2015; Raucher and Ryu 2015; Langel 2019; Foerg and Merkle 2008; Koren and Torchilin 2012; Alvaro et al. 2016; Ruseska and Zimmer 2020). Since the discovery of HIV-1 TAT (Frankel and Pabo 1988; Green and Loewenstein 1988) and *D. Melanogaster* pAntp (Joliot et al. 1991) and their respective shorter fragments $\mathrm {TAT}_{48-60}$ (Vives et al. 1997), $\mathrm {TAT}_{49-57}$ (Park et al. 2002) and  penetratin  (Derossi et al. 1996) as the first CPPs, many other CPPs have been identified. For instance, polyarginines may act as CPPs and perform better as a CPP than TAT derivatives (Wender et al. 2000; Mitchell et al. 2000). Although CPPs vary greatly in sequence, most of them are overall cationic or amphipathic (Milletti 2012). A database called CPPSite has been created to provide a comprehensive overview of peptide sequences reported as CPPs (Gautam et al. 2012; Agrawal et al. 2015).

Several cell-entering mechanisms have been proposed for CPPs (Gasparini et al. 2015; Guidotti et al. 2017; Gestin et al. 2017; Milletti 2012; Kauffman et al. 2015; Raucher and Ryu 2015; Langel 2019; Foerg and Merkle 2008; Koren and Torchilin 2012; Alvaro et al. 2016; Ruseska and Zimmer 2020), but no rational design for a specific pathway or behaviour based on sequence has been achieved to date (Kauffman et al. 2015; Ramaker et al. 2018). In general, positively charged CPPs are accepted to enter cells by sequestering on the often negatively charged cell surface and subsequently undergoing some form of endocytosis (’energy-dependent’ pathways) (Guidotti et al. 2017; He et al. 2013; Kauffman et al. 2015; Gasparini et al. 2015; Poon and Gariépy 2007). However, also energy-independent spontaneous translocation of CPPs has been evidenced (Futaki et al. 2007; Futaki and Nakase 2017; Hirose et al. 2012; Kawamoto et al. 2011; Derossi et al. 1996), but how CPPs achieve this remains a matter of controversy: most proposed mechanisms involve pore formation or transient pore formation or otherwise membrane disruption (Allolio et al. 2018; Herce et al. 2009, 2014). Reconciling such mechanism with the CPPs’ low cytotoxicity has been difficult because pore formation can lead to leakage of cell contents, which is considered inevitably toxic (Kauffman et al. 2015; Hirose et al. 2012). Membrane disruption is a behaviour rather ascribed to another category of membrane-active peptides, antimicrobial peptides (AMPs) (Alvaro et al. 2016; Zasloff 2002; Wimley and Hristova 2011).

It is worth noting that the line between CPPs and AMPs is somewhat blurry (Bobone et al. 2011; Henriques et al. 2006). Hence, CPPs and AMPs have been reviewed together as membrane-active peptides (Alvaro et al. 2016; Splith and Neundorf 2011; Henriques et al. 2006). Some CPPs also show AMP activity (Han and Patil 2000; Bahnsen et al. 2013) and vice versa (Splith and Neundorf 2011; Budagavi and Chugh 2018).

Despite the limited possibilities for rational design and clear classification of CPPs and the controversy over their mechanism of action, it is widely accepted that the guanidinium-bearing side chains of the amino acid arginine play an important role. The majority of CPPs and AMPs is rich in arginine and/or lysine (Gasparini et al. 2015; Guidotti et al. 2017; Gestin et al. 2017; Milletti 2012; Kauffman et al. 2015; Raucher and Ryu 2015; Foerg and Merkle 2008; Koren and Torchilin 2012; Alvaro et al. 2016), including the firstly discovered CPPs TAT-analogs (Vives et al. 1997; Park et al. 2002) and penetratin (Derossi et al. 1996). Arginine residues enable the peptides to recognize negatively charged lipids by electrostatic interactions and formation of bidentate hydrogen bonds (Henriques et al. 2006; Gasparini et al. 2015; Nakase et al. 2008; Kauffman et al. 2015). Furthermore, it has been demonstrated repeatedly that replacing lysine or other amino acids with arginine enhances CPP or AMP activity, or that replacing arginine with lysine diminishes activity or binding ability (Bahnsen et al. 2013; Buschle et al. 1997; Bobone et al. 2011; Mitchell et al. 2000; Wender et al. 2000; Tunnemann et al. 2008; Robison et al. 2016).

The properties of the arginine side chain that likely make for their activity have been reviewed in context of supramolecular chemistry (Gasparini et al. 2015) and energetics (Hristova and Wimley 2011). The dubbed ’arginine magic’ has been ascribed to stronger binding to negative counterions, due to the weaker acidity of the guanidinium cation as compared to the ammonium cation of lysine side chains (Gasparini et al. 2015).

**Fig. 1:**
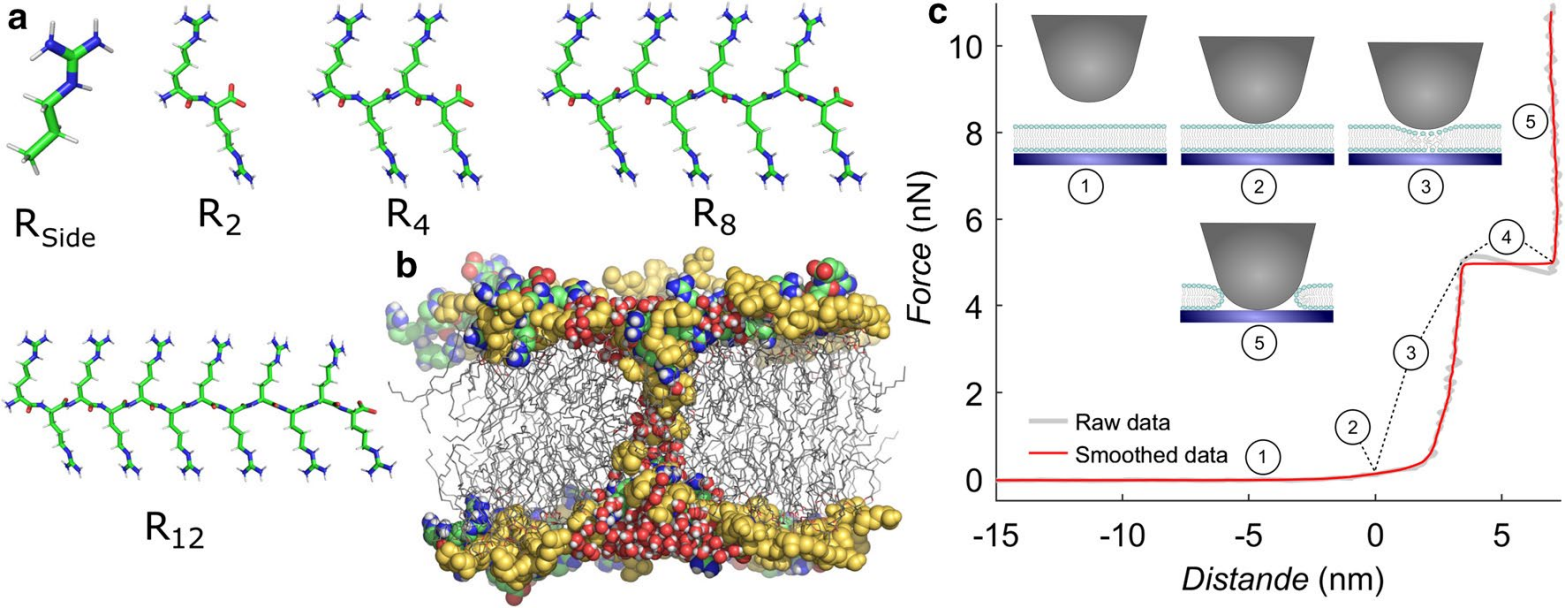
**a** Stick representation of an arginine side chain mimic R$_\mathrm {Side}$ and oligo-arginines R$_\mathrm {2}$, R$_\mathrm {4}$, R$_\mathrm {8}$ and R$_\mathrm {12}$. **b** Simulation snapshot of a POPG membrane with an open pore. Lipid headgroups are represented as yellow spheres, tails as grey sticks. For clarity, only water molecules near the pore are shown as red/white spheres. Arginine-8 is shown as colored green/blue/white/red spheres. **c** Exemplary force vs. distance curve with a breakthrough event, as collected on the atomic force microscope, with illustrations of the cantilever tip and membrane. (1) As the cantilever approaches, its deflection is initially zero. The contact point (2) is usually defined as the point from which cantilever deflection starts increasing. The cantilever tip deforms the bilayer as it exerts more force (3), until at some point it breaks through with a discrete event with two kinks (4). After breakthrough, the cantilever is in contact with the solid support (5). The force at which the breakthrough event occurs is defined as ’yield force’, in our case the average force of plateau (4)

Most previous studies provided mainly qualitative insight into the CPP activity of polyarginines because the outcome of common vesicle leakage or cellular uptake assays is essentially binary. Consequently, only limited quantitative or energetic understanding of the CPP activity of polyarginines has emerged. In addition, it is not well understood why only longer polyarginines are efficient CPPs, whereas short polyarginines such as R$_2$ or R$_4$ are inactive. To close these gaps, we studied pore formation using two quantitative, complementary methods: (1) atomic force microscopy (AFM) on solid-supported lipid bilayers in phosphate buffer and (2) free-energy calculations of pore formation based on all-atom molecular dynamics (MD) simulations (Fig. 1). We focus on model membranes with a simple lipid composition, without any transmembrane potential, and without additional proteins. As such, our membranes are considerably simpler as compared to biological membranes since the latter are crowded with proteins, exhibit complex lipid composition, and may be further influenced by electric potentials. In experiment and in simulation, model membranes were composed of common lipids (POPC, POPE, POPG), which we studied under the influence of arginine oligopeptides (further referred to as $\mathrm {R}_{n}$ or R-peptides) of different lengths or of an arginine side chain mimic (N-propylguanidine or R$_{\text {Side}}$), see Fig. 1a. We included peptides of length 6, 8, 9 and 12, which are known to act as CPPs (Balhorn et al. 2009; Ramaker et al. 2018), and compared their influence on pore formation with that of shorter peptides not known to belong to the class of CPPs ($\mathrm {R}_{2}$, $\mathrm {R}_{4}$). To clarify the role of charged termini during pore formation by $\mathrm {R}_{n}$, we further simulated $\mathrm {R}_{n}$ variants with neutral termini ($\mathrm {R}_{n}^\mathrm {cap}$).

Breakthrough force spectroscopy describes the site-specific formation of holes or pores in thin films such as lipid bilayers on a solid support. The force necessary to achieve a mechanical instability of the film is a measure for the resistance of the membrane against pore formation. Breakthrough events can be detected from indentation force curves as a sudden jump into contact with the solid substrate (Fig. 1c). This method has been used before to study the influence of several parameters on membrane mechanics (Garcia-Manyes and Sanz 2010), such as lipid composition (Künneke et al. 2004; Morandat et al. 2013), ionic strength (Garcia-Manyes et al. 2006) or ionic species (Redondo-Morata et al. 2012), added polymers (Sullan et al. 2013) and temperature (Garcia-Manyes et al. 2005). Because it is difficult to obtain free energies from AFM experiments, and because the interactions of the membrane with the cantilever tip and with the solid support may influence the quantitative results (Butt and Franz 2002), we complemented the experiments with MD simulations of pore formation. To obtain the effect of arginines on the energetics of pore formation, we computed potentials of mean force (PMFs, or ‘free energy profiles’) for the formation of a continuous polar defect across the membranes, both in absence and in presence of $\mathrm {R}_{n}$ or $\text {R}_\text {Side}$ (Fig. 1b).

Our combination of methods provides quantitative insight into the effect of arginines on the resistance of membranes against pore formation. Our experiments show that the impact of long $\mathrm {R}_{n}$ and $\text {R}_\text {Side}$ on pore expansion differs qualitatively, suggesting that not merely the guanidine moieties, but also the polyarginine backbone plays a role in pore formation. Furthermore, our simulations reveal that the charged termini of short oligo-arginines prevent their membrane-destabilizing activity, rationalizing why only longer R_*n*_ are membrane-active, for which the charged termini have smaller relative impact. Finally, our experiments strongly suggest that PC headgroups can protect against pore formation induced by oligo-arginines in membranes with high negatively charged lipid content.

## Methods

### Molecular dynamics simulations: setup and parameters

Simulation systems of 128 lipid molecules plus 50 water molecules per lipid were set up with the MemGen webserver (http://memgen.uni-goettingen.de) (Knight and Hub 2015). Systems of four different lipid compositions were build: 1-palmitoyl-2-oleoyl-*sn*-glycero-3-phosphoglycerol (POPG), 1-palmitoyl-2-oleoyl-*sn*-glycero-3-phosphoethanolamine (POPE), 1-palmitoyl-2-oleoyl-*sn*-glycero-3-phosphocholine (POPC), as well as a 1:1 mixture of POPG and POPE. After energy minimization with the steepest descent algorithm, each system was equilibrated until the box dimensions and the potential energy were fully converged.

Next, arginine side chains $\mathrm {R}_\mathrm{Side}$ as well as polyarginines composed of two (R$_2$), four (R$_4$), or eight arginine residues (R$_8$) were added to the system. In addition, to test the influence of charged termini, we further added R$_2$ or R$_4$ variants with electroneutral termini, denoted R$_2^\mathrm {cap}$ and R$_4^\mathrm {cap}$, by adopting deprotonated N- and protonated C-termini. Side chains and oligo-arginines were added with a lipid-to-residue ratio of 2:1, that is, we added 8 R$_8$, 16 R$_4$/R$_4^\mathrm {cap}$, 32 R$_2$/R$_2^\mathrm {cap}$, or 64 $\mathrm {R}_\mathrm{Side}$. Moreover, to test the effect of arginine concentration, we set up additional systems with 32 $\mathrm {R}_\mathrm{Side}$. All systems were neutralized by adding either sodium or chloride ions, depending on the overall charge of the system. Before forming a pore into these membranes (see below), the systems were equilibrated for another 20 ns.

Simulations were carried out with the GROMACS simulation software, version 2016 (Abraham et al. 2015). Lipid interactions were described by the GROMOS-CKP united-atom force field (Piggot et al. 2011, 2012), and the SPC water model was used (Berendsen et al. 1981). Arginine derivatives were modeled with the Gromos54a7 parameters (Schmid et al. 2011), which are compatible with the GROMOS-CKP lipid parameters. Lipid topology files were taken from Lipidbook (Domański et al. 2010). Several measures were taken to allow an integration time step of 4 fs and, thereby, to reduce the considerable computational cost of the simulations reported here. First, to reduce the frequency of bond vibration in the amine groups of POPE, we increased the hydrogen mass to 4 amu and, to maintain the overall mass constant, reduced the nitrogen mass to 5 amu. Second, we constrained the C–O–H angle of the two hydroxyl groups of POPG to their average values of 0.199 nm and 0.201 nm, respectively, obtained from a non-constrained simulation with a 2 fs time step. This setup corresponds to the common treatment of hydroxyl groups if hydrogen atoms are treated by the GROMACS software as virtual sites. We validated that these modifications have only a marginal effect (if any) on the area per lipid and on the hydrogen bonding of POPG lipids (supporting Table 2). Third, hydrogen atoms of arginine were described as virtual interaction sites. Bonds and angles of water were constrained with the SETTLE algorithm (Miyamoto and Kollman 1992). Other bonds were constrained with P-LINCS (Hess 2008). The temperature was controlled at 323 K using velocity rescaling ($\tau = 0.5$ ps) (Bussi et al. 2007). The pressure was controlled at 1 bar using a semi-isotropic weak coupling scheme ($\tau =1$ ps) (Berendsen et al. 1984). Although weak coupling does not yield a well-defined ensemble, we used it here owing to its numerical stability. Electrostatic interactions were calculated using the particle-mesh Ewald (PME) method (Darden et al. 1993; Essmann et al. 1995). Dispersion interactions and short-range repulsion were described by a Lennard–Jones potential with a cutoff at 1 nm.

### Potential of mean force calculations of pore formation

Potentials of mean force (PMFs) of pore formation were computed for pure-lipid systems, as well as for the POPG systems with $\mathrm {R}_\mathrm{Side}$, R$_4$, R$_4^\mathrm {cap}$, and R$_8$. The PMFs were computed along a recently developed reaction coordinate, here referred to as ‘chain coordinate’ $\xi _\mathrm {ch}$ (Hub and Awasthi 2017; Awasthi and Hub 2019). In brief, $\xi _\mathrm {ch}$ is defined with a membrane-spanning cylinder that is decomposed into slices with a thickness of typically 1Å. Then, $\xi _\mathrm {ch}$ is approximately given by the fraction of slices that are occupied by polar heavy atoms, in this work taken as oxygen atoms of water and lipid phosphate groups. As such, $\xi _\mathrm {ch}$ is unit-less and probes the degree of connectivity of a polar transmembrane defect: a value of $\xi _\mathrm {ch} \approx 0.25$ indicates the flat membrane, with only few cylinder slices at the two headgroup regions filled by polar atoms. In contrast, a large value of $\xi _\mathrm {ch} \gtrsim 0.85$ indicates a continuous, membrane-spanning polar defect. Critically, the membrane-spanning cylinder does not take a fixed lateral position in the membrane plane. Instead, the lateral position of the cylinder is dynamically defined so it can ‘follow’ the polar defect while it travels along the membrane plane. This property excludes that the system moves along the reaction coordinate by shifting the defect laterally out of the cylinder, which would lead to undesired hysteresis effects during PMF calculations (Awasthi and Hub 2016). The reaction coordinate was implemented into an in-house modification of GROMACS 2016, which is available from the authors upon request.

PMFs of pore formation were computed using umbrella sampling (Torrie and Valleau 1974), similar to previous work (Ting et al. 2018; Awasthi et al. 2019). The initial frames for umbrella windows were taken from slow-growth pulling simulations along $\xi _\mathrm {ch}$ over 46 ns. The cylinder had a radius of 1.2 nm and contained 30 slices with a thickness of 0.1 nm. The parameter $\zeta$, specifying the fraction to which a slice is filled upon the addition of the first polar atom, was set to 0.75. We used 24 umbrella windows restrained to the following, non-uniformly distributed $\xi _\mathrm {ch}$ positions: 0.065–0.625 in steps of 0.08 using a force constant of 5000 kJ/mol, and 0.7– in steps of 0.02 using a force constant of 10,000 kJ/mol. For systems with arginine or arginine side chains, each window was simulated for 1 $\upmu$s, where the first 0.5 $\upmu$s were omitted for equilibration. For pure-lipid systems, each window was simulated for 150 ns, where the first 20 ns were omitted for equilibration. Here, the temperature was controlled using a stochastic dynamics integration scheme ($\tau = 0.5$ ps) (Gunsteren and Berendsen 1988). All other parameters were chosen as described above. The PMFs were computed with the weighted histogram analysis method (WHAM) (Kumar et al. 1992), as implemented in the gmx wham module of GROMACS (Hub et al. 2010). Statistical errors were estimated using 50 rounds of Bayesian bootstrapping of histograms (Hub et al. 2010). Before computing the average and standard deviation of the PMFs obtained from bootstrapped histograms, the PMFs were defined to zero at the PMF minimum ($\xi _\mathrm {ch} \approx 0.25$).

### Experimental materials

1-Palmitoyl-2-oleoyl-*sn*-glycero-3-phosphocholine (POPC), 1-palmitoyl-2-oleoyl-*sn*-glycero-3-phospho-(1’-rac-glycerol) (sodium salt, POPG) and 1-palmitoyl-2-oleoyl-*sn*-glycero-3-phosphoethanolamine (POPE) were bought as 10 mg/ml solutions in chloroform from Avanti Polar Lipids and used as supplied. The lipid-tail fluorophore $\upbeta$-BODIPY 500/510 $\mathrm {C} _{12}$-HPC (2-(4,4-Difluoro-5-Methyl-4-Bora-3a,4a-Diaza-$s$-Indacene-3-Dodecanoyl)-1-Hexadecanoyl-$sn$-Glycero-3-Phosphocholine, further denoted as Bodipy or BP) was obtained from ThermoFisher Scientific. It was dissolved to 0.1 mg/mL in HPLC-grade ethanol-stabilised chloroform and stored in a brown glass vial at – 24 $^\circ$C. All buffers were prepared using ultrapure water with a resistivity of $18.2\,\hbox {M}\varOmega \,\hbox {cm}$, as obtained from a Milli-Q Advantage A10 Ultrapure Water Purification System equipped with a MilliPak®Express 40 filter with $0.22\,{\upmu }\hbox {m}$ pore size (Merck Millipore). 1-propyl guanidine (N-propylguanidine, $\mathrm {R}_{\mathrm {Side}}$) was bought from SelectLab Chemicals. All peptides were ordered from Bachem. Diarginine (H-Arg-Arg-OH, $\mathrm {R}_{2}$) and was bought as an acetate salt. Hexa-arginine ($\mathrm {R}_{6}$), octa-arginine ($\mathrm {R}_{8}$), and dodeca-arginine ($\mathrm {R}_{12}$, obtained via custom order) were obtained as a trifluoroacetate salt. Each R-derivative was dissolved to a 1 M or 0.5 M solution in measuring buffer (50 mM $\mathrm {Na}_{2}\mathrm {HPO}_{4}$, 0.1 mM EDTA, pH 6.8) and 100 mM and 200 mM stock solutions were diluted from this solution with the same buffer. All R-derivative stock solutions were stored at $-24\,^{\circ }$C. P-type, boron-doped silicon wafers of 725 $\upmu$ m thickness with a 100 nm $\mathrm {SiO}_{2}$-layer and a resistivity of 1–50 $\varOmega$ cm were used as solid supports and obtained from Active Business Company.

### Preparation of lipid vesicles

Vesicles were created from dried lipid films by hydration. Glass tubes were thoroughly rinsed with water, ethanol p.a. and HPLC-grade chloroform before deposition of lipid films. Lipids and Bodipy stock solutions in chloroform were combined in a glass tube in the desired molar ratio for a 0.5 mg lipid film. Molar ratios were either POPC:BP 99:1 (further denoted POPC) or POPC:X:BP 49.5:49.5:1, X being POPE or POPG (further denoted as POPC:POPG/E 1:1) After all necessary stock solutions were mixed, the walls of the glass tube were rinsed with 150 $\upmu$L of chloroform, to gather all lipids at the bottom of the tube. Solvents were removed in a water bath of 35 $^{\circ }$C under nitrogen stream and subsequent exposure to vacuum at 55  $^{\circ }$C overnight. Lipid films were covered and stored at 4 $^{\circ }$C for two weeks at most.

Before use, lipid films were hydrated with 600 $\upmu$L of measuring buffer and swollen for 10 min (at room temperature for POPG-containing lipid films, in a 55 $^{\circ }$C water bath for other films). Subsequently, they were suspended in the glass tube to form multilamellar polydisperse vesicles, and transferred to a 1.5 mL microcentrifuge tube. Small unilamellar vesicles (SUVs) were obtained by sonication for thirty minutes in a Bandelin Sonopuls HD2070 sonifier equipped with a BR30 beaker resonator and an EH3 reaction cup holder (Bandelin Electronic, Berlin, Germany), at 65% power and a 4 $\times$ 10% pulsed cycle. Vesicles composed of mainly POPC or a mixture of POPC and POPE were additionally pressed over a 0.45 $\upmu$m PTFE syringe filter (diameter 13 mm, VWR International).

### Preparation of solid-supported membranes

Directly before use, silicon wafers were cut to ca. 7 mm $\times$ 22 mm, thoroughly rinsed with water and ethanol p.a., and dried under nitrogen stream. Wafers were cleaned with an oxygen plasma generated in a Diener Zepto plasma cleaner (Diener Electronic) as follows: 15 min of oxygen flow at 25 mbar, followed by a plasma process of 5.5 min at 25 mbar and 60% power. Subsequently, the wafers were mounted in home-built Teflon measuring chambers and hydrated with $350\,{\upmu }\hbox {L}$ of spreading buffer (20 mM trisodium citrate, 50 mM KCl, 0.1 mM EDTA, 0.1 mM $\mathrm {NaN}_{3}$, pH 4.8) immediately after plasma treatment. 250 $\upmu$L of vesicle solution (prepared as described above) was added and a bilayer formed over two hours at room temperature. 400 $\upmu$L of measuring buffer was added, and remaining vesicles were rinsed off with $6\,\times \,1$ mL of measuring buffer. 600 $\upmu$L of measuring buffer was added again to yield a sample volume of 1600 $\upmu$L.

### Breakthrough force spectroscopy

AFM experiments were performed with a JPK NanoWizard III or NanoWizard IV AFM (JPK Instruments). Bruker MLCT silicon nitride microlevers with silicon nitride tips were used in all AFM experiments (tip radius 20–60 nm, Bruker France S. A. S.) Triangular probe D or E on a chip was used exhibiting spring constants of ca. 0.060 or 0.200 N/m, respectively. Spring constants were calibrated by using the thermal noise method combined with determination of the ratio of PSD-voltage to piezo movement (Butt et al. 2005; Hutter and Bechhoefer 1993). All breakthrough force experiments were performed with 1 $\upmu$m/s approach and retract velocity. At least 750 breakthrough force curves were collected of the solid-supported lipid bilayer without the R-derivative. Maximum force and retract distance were chosen according to the sample. Then R-derivative was added from a stock solution at least 100$\times$ the desired concentration, and again more than 750 breakthrough force curves were collected. Basic processing of the force curves was performed using JPKPSM data processing software (version 6.0.40) to obtain baseline-corrected force-distance curves with determined contact point (as single files). Automatic analysis of breakthrough events was performed using a self-written Matlab script which analyses the first derivative of the force curve above a preset threshold. The yield force *F* was thus defined as the mean force of all points in the detected breakthrough event. At least two experiments were performed for each combination of lipid mixture and R-derivative. It was observed that the median force detected differed per experiment and per cantilever used. This was attributed to possible differences in tip radius of individual cantilevers, which influences the breakthrough force distribution and penetration depth before the event occurs (Saavedra et al. 2020). Hence, to combine datasets from different experiments, data were normalized for the median of the yield force obtained on the lipid mixture without R-derivative. Fits to Butt and Franz’s continuum nucleation model (Butt and Franz 2002; Franz et al. 2002) were achieved by a trust-region method implemented in Matlab.

## Results

### Experimental assessment of pore formation

Solid-supported membranes prepared from vesicle spreading were subject to indentation experiments with conventional AFM tips until rupture events were detected as instabilities in the approach curve. These rupture events occur stochastically and characterize the stability of membranes towards formation of holes. The yield forces reflect both the change in surface free energy as well as the line tension of the pore arising due to the exposure of alkyl chains to the aqueous surrounding after punching a hole in the bilayer structure. The analogy to hole formation due to the action of peptides is used in this study to quantify the action of R$_{\text {Side}}$ and R-peptides, which potentially destabilize the membrane leading eventually to leaky lipid bilayers. The external force permits to move slow processes (low rates) into an experimentally accessible time window by lowering the activation barrier in the same manner as in unfolding proteins and rupture of individual bonds (Oelkers et al. 2016).

**Fig. 2:**
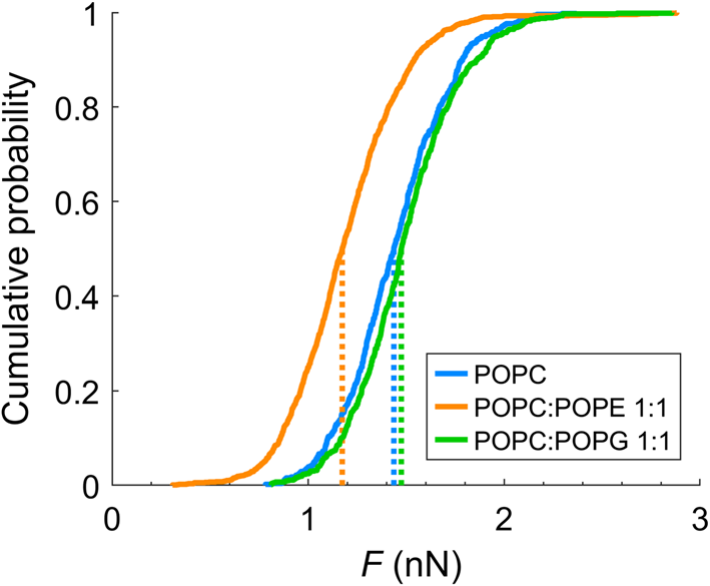
Empirical cumulative distribution function of yield force *F* as obtained on the three investigated bilayers using the same cantilever. Medians of data distributions are indicated with dotted lines

First, we studied the mechanical stability of neat lipid bilayers. In Fig. 2, a cumulative histogram of the yield forces *F* measured for pure POPC, POPC:POPE 1:1 and POPC:POPG 1:1 is shown. All data were obtained with the same cantilever/tip ensuring the highest possible comparability of their mechanical properties. Yield forces can usually vary from cantilever to cantilever since the variability of the tip radius has considerable influence on the measured yield force (Butt et al. 2005; Saavedra et al. 2020).

**Fig. 3:**
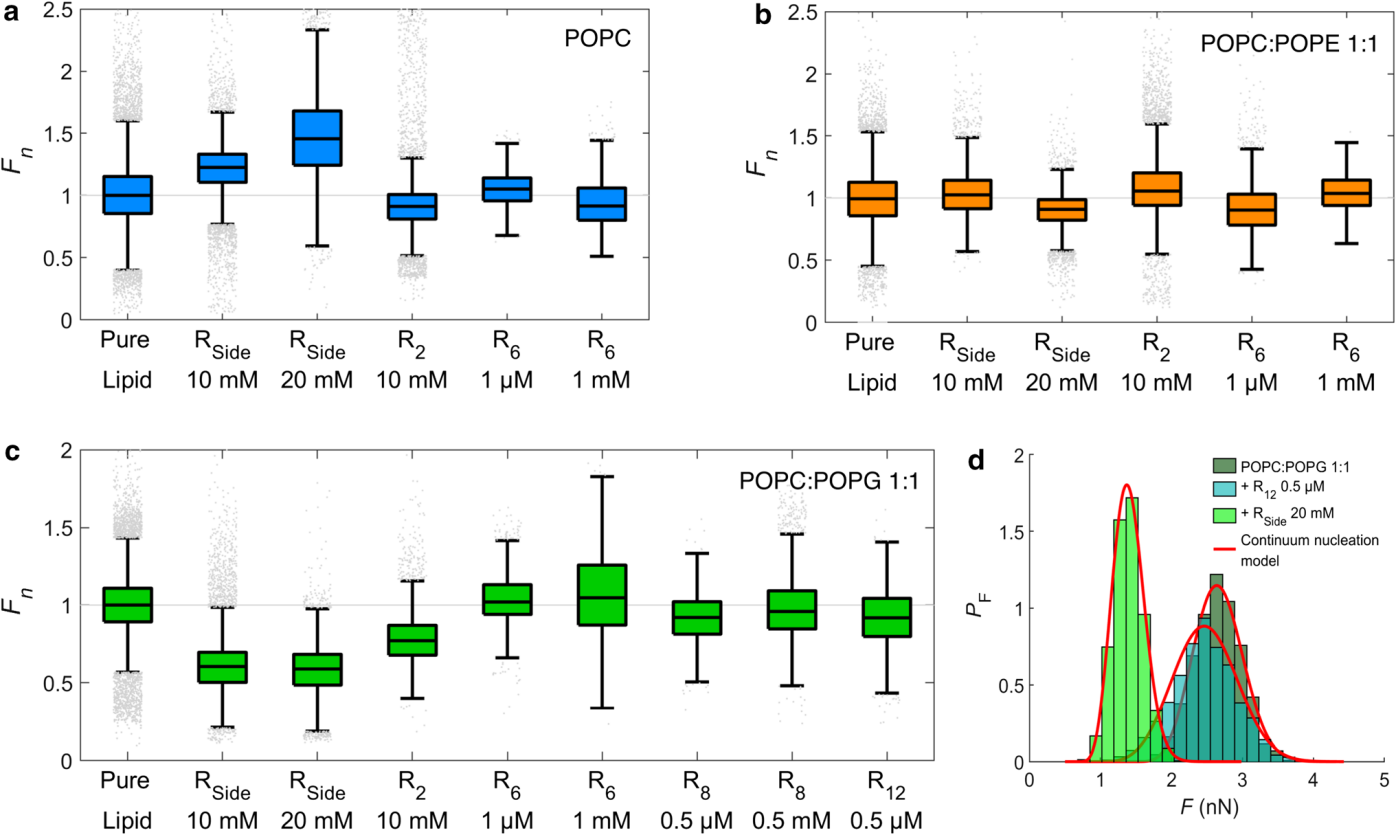
Boxplots of normalized yield forces $F_n$ of membranes varying in composition after incubation with different R-derivatives. Normalization was performed for the median of the yield force obtained in the absence of R-derivatives for each experiment. Each boxplot contains combined data from at least two independent experiments. The bottom and top edge of the boxplots indicate the 25th and 75th percentile of each data set, and the line dividing the box indicates the median. The upper and lower whisker represent approximately 2.7 standard deviations higher or lower than the mean, respectively. Outliers (points beyond 2.7 standard deviations from the mean) are shown as grey points. **a** POPC; **b** POPC:POPE 1:1; **c** POPC:POPG 1:1. **d** Normalized histograms of yield force results of POPC:POPG 1:1 and a combination with $\mathrm {R}_\mathrm{Side}$ and $\mathrm {R}_{12}$, respectively, all probed with the identical cantilever. The probability density function of each data histogram was fitted (red lines) to a continuum nucleation model as introduced by Loi et al. (Loi et al. 2002), to obtain line tension ${\varGamma }$ and spreading pressure *S*

Despite the influence of tip-radii, yield forces obtained for all lipid mixtures are of a similar order of magnitude, with POPE-containing membranes displaying a significantly lower rupture force. Over the course of all experiments, median yield forces of around 1–5 nN were obtained (exemplary data in supporting information). For the sake of comparability, we normalized the yield force to the initial yield force of the bilayer in the absence of R-derivatives. Figure 3 summarizes the normalized yield force $F_{n}$ for POPC (a), POPC:POPE 1:1 (b) and POPC:POPG 1:1 (c). Employed concentrations of R-derivatives were chosen in the order of magnitude of the dissociation constants on POPC:POPG 1:1 as determined by reflectometric interference spectroscopy (data in supporting information).

Interestingly, breakthrough force spectroscopy shows that R$_{\text {Side}}$ facilitates pore formation in PG-containing bilayers the most. Addition of soluble R$_{\text {Side}}$ to bilayers with a composition of POPC:POPG 1:1 lowers the yield force significantly by about 40% (Fig. 3c) compared to bilayers in the absence of R-derivatives.

In POPC-membranes, R$_{\text {Side}}$ had a rather opposite effect on the yield force. The force to penetrate the POPC bilayer increased after addition of R$_{\text {Side}}$ in a concentration-dependent manner. This is, however, most likely due to a slight increase in pH. The addition of large quantities of R$_{\text {Side}}$ shifts the pH slightly to larger values albeit working in buffered conditions. Hence, to discriminate between (1) effects from R$_{\text {Side}}$-lipid interactions from (2) effects from a modified pH, experiments were performed in absence of R$_{\text {Side}}$ but with an exchange of measuring buffer from pH 6.8 to 7.4, corresponding to the estimated order of pH change upon adding R$_{\text {Side}}$ (data in supporting information). For both POPC membranes, the increased pH led to an increased yield force, suggesting that the R$_{\text {Side}}$ effect on POPC was indeed due to an increased pH. For POPC:POPG 1:1, the same effect occurred upon increasing pH, which strongly suggests that the lowering of the mean yield force in presence of R$_{\text {Side}}$ is not caused by the pH change but rather by a significant and substantial destabilization of the bilayer due to R$_{\text {Side}}$-lipid interactions.

Addition of oligo-arginines, however, did not result in substantial lowering of yield forces. Importantly, we adapted the added concentrations of oligomeric arginine derivatives to the corresponding $K_d$ values obtained from optical measurements. Thereby, we could compare the membrane activity of the different compounds. Neither addition of higher concentrations (up to the millimolar range, which is a factor 100 higher than the dissociation constant) for R$_{\text {6}}$ and R$_{\text {8}}$ led to noticeable changes of the breakthrough forces, nor did administration of a considerably longer poly-arginines R$_{\text {12}}$ change the stability of the bilayer. The lack of destabilization of the membrane suggests that if poly-arginines cause pore formation in membranes, they do so via a different mechanism than a lone guanidine moiety R$_{\text {Side}}$.

How do yield forces on lipid bilayers relate to the free energy of hole formation in membranes? Butt and coworkers essentially devised two models that serve to explain the experiment, a stochastic molecular model, which is similar to Bell’s approach used to describe single molecule rupture events, and a continuum nucleation model that we will adapt in the following (Butt and Franz 2002; Loi et al. 2002). The continuum nucleation model considers the failure of a thin film (such as a membrane) under a cantilever tip being an activated process under an externally applied force. Thermal fluctuations eventually form a tiny circular hole (radius $r_\mathrm {h}$) in the bilayer under the tip. The energy *U* of such a hole is given by (Butt and Franz 2002):

1 $$\begin{aligned} U= 2 \pi r_\mathrm {h}\varGamma + \pi r_\mathrm {h}^2 \left( S-\frac{F}{2\pi R}\right) , \end{aligned}$$

where *R* is the cantilever tip radius (typically 20 nm), *F* is the applied force, and $\varGamma$ is the line tension. *S* is the change in the interface free energy when removing a membrane patch between tip and substrate, also referred to as ’spreading pressure’. The spreading pressure *S* encompasses all relevant interfacial free energies:

2 $$\begin{aligned} S=\gamma _\mathrm {TL}+\gamma _\mathrm {SL}-\gamma _\mathrm {TM}-\gamma _\mathrm {SM}, \end{aligned}$$

where the subscripts T, L, M, and S denote tip, liquid (the aqueous environment), membrane, and substrate, respectively. *S* is most likely to be a positive value since covering the solid surfaces with membrane is energetically less costly than a solid surface in contact with the aqueous environment. In other words, formation of the bilayer relies on a positive spreading pressure. Larger values for *S* are due to smaller surface energies $\gamma _\mathrm {SM}$ and $\gamma _\mathrm {TM}$ and thus require larger external forces to create holes in the bilayer. Smaller values for *S* can be interpreted also as a better solubility of membrane patches.

Butt and coworkers used an elastic foundation model to show that once the external force exceeds the spreading force $2\pi R S$, the energy $U(r_\mathrm {h})$ passes through a maximum at critical radius ($r_\mathrm {h} = r_\mathrm {c} = 2 \pi R \varGamma /(F-2 \pi R S)$) (Butt and Franz 2002):

3 $$\begin{aligned} \triangle U=\frac{2\pi ^{2}\varGamma ^{2}R}{F-2\pi RS}. \end{aligned}$$

Based on Bell’s theory, the following probability density distribution function for the yield force can be obtained:

4 $$\begin{aligned} P_{F}=\left| \frac{d}{dF}\left( 1-\exp \left( -\frac{A}{K\nu }\intop _{2\pi RS}^{F}1-\exp \left( \frac{2\pi ^{2}\varGamma ^{2}R}{(F-2\pi RS)k_{b}T}\right) dF\right) \right) \right| \end{aligned}$$

with $\nu$ the approach velocity of the cantilever, *K* its spring constant, and *A* a frequency factor for ’attempts’ at breaking through the membrane, which is limited by the cantilever’s main resonance frequency (in our case 3 kHz, as determined experimentally). $k_{b}$ and *T* denote Boltzmann’s constant and temperature, respectively. $P_F$ can be fitted to a population of yield force data in order to obtain values for *S* and $\varGamma$.

Figure 3d shows the corresponding fit to histograms of yield forces obtained on a POPC:POPG 1:1 bilayer before and after addition of either R$_{\text {12}}$ or R$_{\text {Side}}$, using the same cantilever. The obtained values for *S* and $\varGamma$ are provided in Table 1. In case of R$_{\text {Side}}$, both $\varGamma$ and *S* are reduced with respect to the pure lipid bilayer, which can be expected for a general destabilization of the membrane. Conversely, for R$_{\text {12}}$, the line tension increases with respect to the pure lipid, while *S* is reduced. Taken together, both R$_{\text {Side}}$ and poly-arginines display smaller spreading pressures indicating that the interfacial energy $\gamma _\mathrm {SM}$ and $\gamma _\mathrm {TM}$ between membrane and solid support increases. This leads to a smaller overall adhesion energy. It is, therefore, conceivable that the dissolved membrane patch after formation of the hole is stabilized in solution by the presence of *R*-derivatives. Critically, because the spreading pressure *S* is influenced by the interactions of the solid support and the tip, it is difficult to tell what the reduced *S* implies for a biologically more relevant, free-standing membrane. Our measured line tension $\varGamma$, however, likely holds for a free-standing membrane; as such, the opposite effects of R$_{\text {12}}$ and R$_{\text {Side}}$ on $\varGamma$ suggest that R$_{\text {12}}$ and R$_{\text {Side}}$ also influence biologically relevant membranes in different manners.

**Table 1:** Line tension ${\varGamma }$ and spreading pressure *S* as obtained by fitting a continuum nucleation model to yield force data of a POPC:POPG 1:1 bilayer and its combination with $\mathrm {R}_{12}$ and $\mathrm {R}_{\mathrm {Side}}$, respectively

	POPC:POPG 1:1	+ $\mathrm {R}_{12}$ 0.5 $\upmu$M	+ $\mathrm {R}_{\mathrm {Side}}$ 20 mM
${\varGamma }\times 10^{-3}$ ($\mathrm {nN}$)	6.93 ± 0.02	8.49 ± 0.04	4.91 ± 0.01
${S}\times 10^{-3}$ ($\text{Nm}^\text{-1}$)	9.49 ± 0.06	3.65 ± 0.01	4.30 ± 0.02

**Fig. 4:**
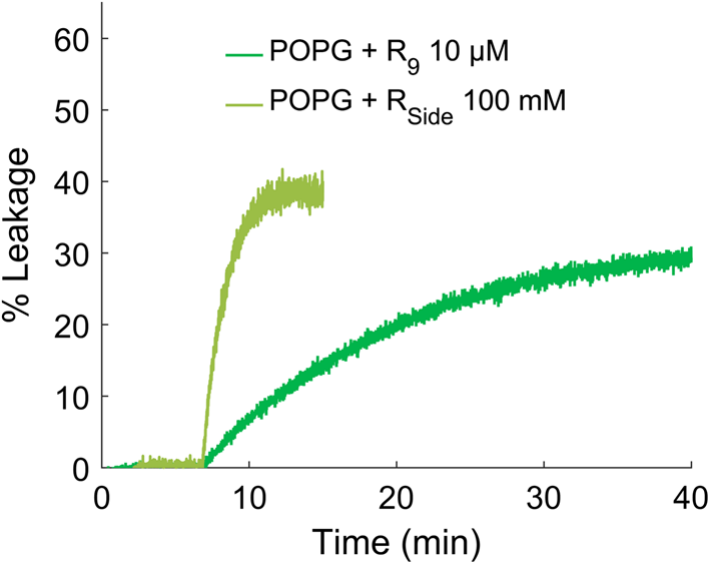
Exemplary leakage assays of sulforhodamine B from pure POPG vesicles. With lower concentrations of $\mathrm {R}_\mathrm {Side}$ or $\mathrm {R}_{9}$ than indicated or with POPC:POPG 1:1, no leakage was achieved (data not shown)

The different behavior of oligoarginines and R$_{\text {Side}}$ motivated us to carry out two additional experiments: a leakage assay to confirm that indeed holes are formed in a lipid bilayer and adsorption measurements using reflectometric interference spectroscopy (RIfS, methods in supporting information) to monitor thickness changes due to the action of R$_{\text {Side}}$ and R-peptides. Figure 4 shows that both poly-arginines (R$_{\text {9}}$) and R$_{\text {Side}}$ can induce partial leakage in pure POPG vesicles. The required concentration is high and no leakage was observed for POPC/POPG membranes (data not shown).

**Fig. 5:**
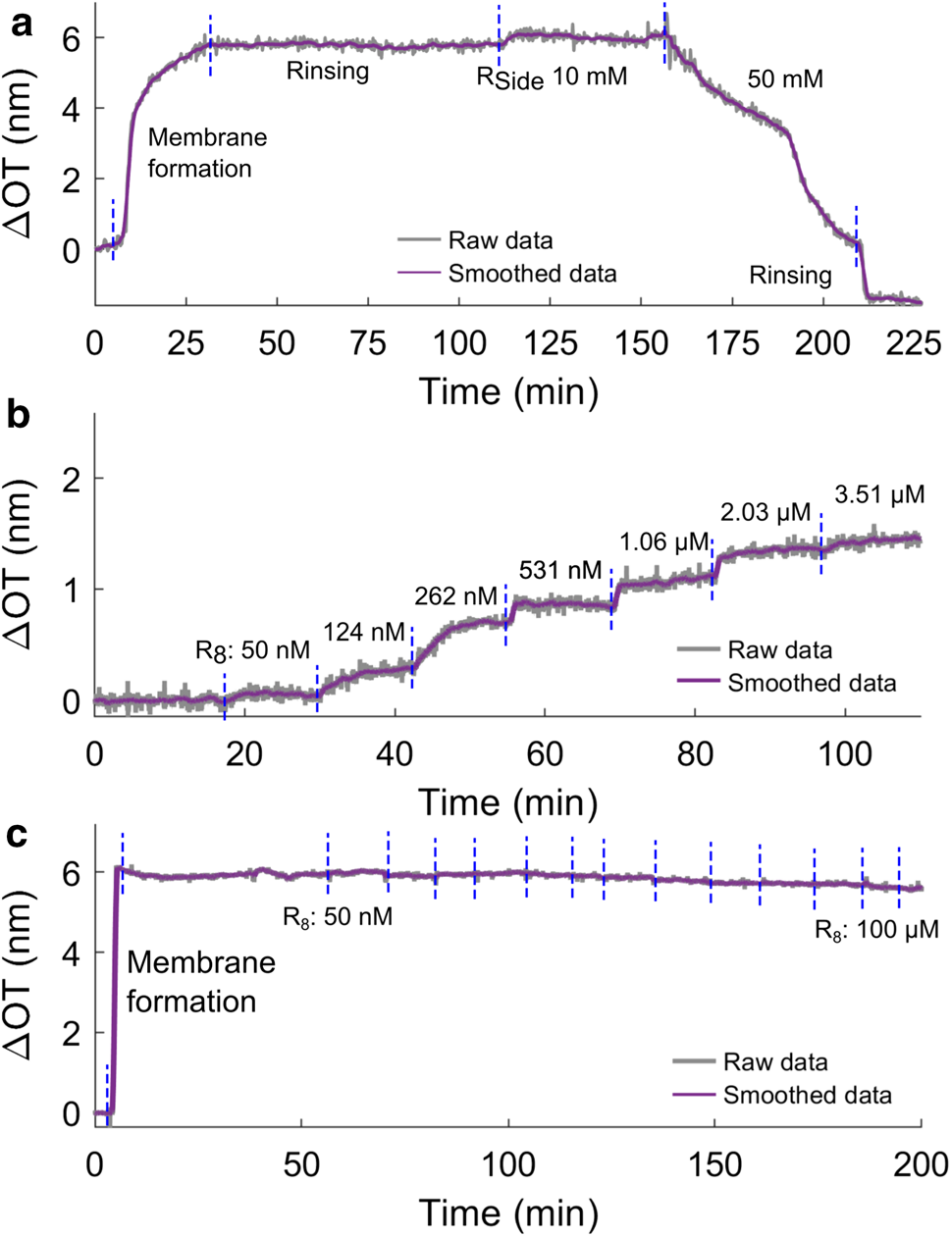
Exemplary RIfS experiments illustrating the different behavior of $\mathrm {R}_\mathrm{Side}$ and R-oligopeptides. Change in bulk concentration is indicated by blue dashed lines. **a** $\mathrm {R}_\mathrm{Side}$ added to a POPC:POPG 1:1 membrane, the membrane was dissolved at the second addition of peptide as the membrane thickness decreases; (**b**/**c**) step-wise increase in $\mathrm {R}_{8}$-concentration in the presence of a POPC:POPG 1:1 bilayer (**b**) and POPC (**c**). No significant increase in optical thickness and hence no binding is observed in the absence of negative charges on the bilayer

RIfS data reveal that the mode of action might indeed differ. While the polyarginines form a detectable layer on the membrane on the order of 1 nm, no such effect is observed after addition of R$_{\text {Side}}$ to the bilayer. In contrast, the membrane destabilizes in the presence of R$_{\text {Side}}$ and eventually disintegrates (Fig. 5a). Additionally, formation of a peptide layer was not detected on pure POPC-membranes, confirming that R-peptides mostly interact with negatively charged lipids.

The finding that polyarginines form a layer on top of POPG-containing solid-supported membranes is further confirmed by force curves in the region of the contact point: addition of higher concentrations of R$_{\text {6}}$ or a longer polyarginine R$_{\text {8}}$ or R$_{\text {12}}$ resulted in a consistent jump-to-contact event (Supporting Information). This is due to attractive interactions between the negatively charged tip and the now positively charged bilayer. Taken together, we infer from our experimental data that a carpet of poylarginines forms on POPG-containing bilayers, while integration of $\mathrm {R}_\mathrm{Side}$ first destabilizes the lipid bilayer by forming pores and eventually solubilizes the membrane.

### MD simulations

To understand the effects of R-derivatives in atomic and energetic detail, we carried out MD simulations of membranes composed of POPC, POPE, and POPG in the presence and absence of R-derivatives. Visual inspection of the simulations revealed that, in simulations with POPC or POPE, R-derivatives do not preferentially interact with the lipid but remain mainly solvated in water. In contrast, R-derivatives bind into the headgroup region of the anionic POPG membrane. This observation is in line with our force spectroscopy data that revealed R-derivative effects only for anionic but not for zwitterionic membranes. However, we did not observe a layer of R$_{\text {8}}$ on top of the membrane surface during simulation, at variance with the experiments on solid support.

#### R$_\mathrm {Side}$ and R$_8$ but not R$_4$ reduce free energies of pore formation in POPG

**Fig. 6:**
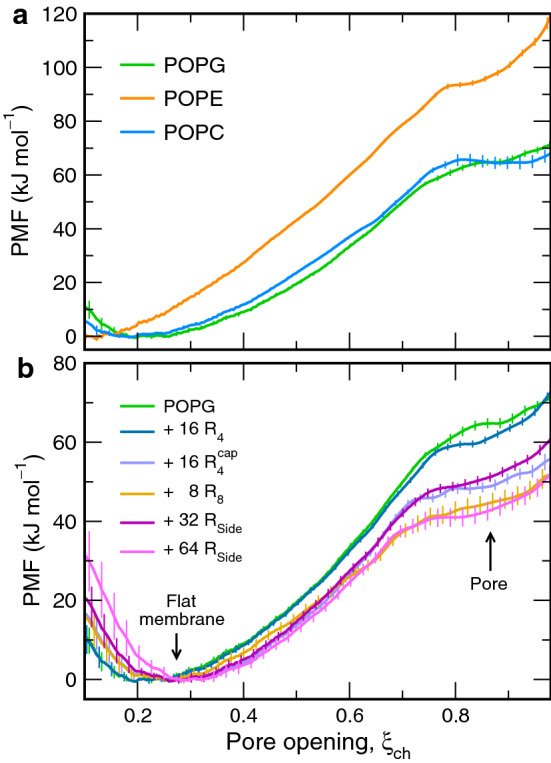
PMFs of pore formation across **a** pure-lipid membranes composed of POPG, POPE, or POPC (see legend) and **b** POPG membranes containing poly-arginine or arginine side chains

We computed potentials of mean force (PMFs), also referred to as ’free energy profiles’, to obtain the free energy cost $\varDelta G_\mathrm {pore}$ for forming a polar defect over the membrane. Figure 6 presents the PMFs of pore formation with purely lipids (Fig. 6a) and with POPG upon the addition of R-derivatives (Fig. 6b). In the PMFs, the global minimum at $\xi _\mathrm {ch} \approx 0.25$ corresponds to the flat, unperturbed membrane, and the plateau at $\xi _\mathrm {ch} > 0.8$ to the state with a continuous transmembrane pore, as visualized in Fig. 1b. A lower value of the PMF at the plateau indicates a membrane that is more prone to form pores, i.e., a destabilization of the flat membrane. For pure POPG or pure POPC, we find $\varDelta G_\mathrm {pore}$ values of approx. 65 kJ/mol (Fig. 6a), which is in reasonable agreement with values of 60 and 77 kJ/mol that we found previously for POPG and POPC, respectively, with the Charmm36 lipid force field at a lower temperature of 300 K as compared to 323 K used here (Ting et al. 2018). For POPE, the PMFs suggest an increased $\varDelta G_\mathrm {pore}$ of $\sim$95 kJ/mol. Overall these free energies characterize membranes that are reasonably stable against pore formation.

These $\varDelta G_\mathrm {pore}$ values can be translated into the free energy $\varDelta G_\mathrm {pore}^\mathrm {exp}$ for forming a pore in a experimentally relevant membrane of area $A_\mathrm {exp}$. Because (1) the probability for a pore under simulation conditions is $[1+\mathrm {exp}(\varDelta G_\mathrm {pore}/k_b T)]^{-1} \approx \mathrm {exp}(-\varDelta G_\mathrm {pore}/k_b T)$ and (2) the probability of finding a pore is proportional to the membrane area, we have

5 $$\begin{aligned} \varDelta G_\mathrm {pore}^\mathrm {exp} = \varDelta G_\mathrm {pore} - k_b T \ln (A_\mathrm {exp}/A_\mathrm {sim}), \end{aligned}$$

where $A_\mathrm {sim} \approx 45$ nm$^2$ is the membrane area in simulation. As a numerical example, for a giant unilamellar vesicle (GUV) with radius of 10 $\upmu$m, $\varDelta G_\mathrm {pore} = 65$ kJ/mol implies $\varDelta G_\mathrm {pore}^\mathrm {exp} = 22$ kJ/mol, indicating a tiny probability for an open pore of $\sim$0.01% (per GUV). For a black lipid membrane on a hole with diameter of 1 mm, however, Eq. 5 implies a $\varDelta G_\mathrm {pore}^\mathrm {exp}$ value of only 6 kJ/mol, indicating a significant probability of $\sim$8% for an open pore.

As shown in Fig. 6b, adding 32 or 64 R$_\mathrm {Side}$ to the POPG membrane stabilizes the open pore in a concentration-dependent manner by up to 22 kJ/mol (Fig. 6b, magenta curves). For the above-mentioned numerical example with a GUV of radius 10 $\upmu$m, that reduction would imply $\varDelta G_\mathrm {pore}^\mathrm {exp} = 0$ kJ/mol, indicating a high probability for the formation of a leaky pore in GUVs. Hence, addition of R$_\mathrm {Side}$ greatly facilitates pore formation over POPG membranes, which is compatible with (1) the reduced breakthrough forces across POPG-containing membranes in the presence of R$_\mathrm {Side}$ in our AFM experiments and (2) with our leakage assays. To quantify the effect of polyarginines on pore formation, we computed the PMFs also in presence of 16 R$_4$ or with 8 R$_8$ peptides. Interestingly, the PMFs imply that R$_8$ but not R$_4$ stabilizes pores (Fig. 6b green and yellow curves), rationalizing in energetic terms why only long but not short R$_n$ are membrane-active.

#### Charged peptide termini counteract effects of guanidium moieties

What makes R$_8$ membrane-active and R$_4$ membrane-inactive? Because we simulated a high concentration of R$_4$ and R$_8$ on a small membrane patch, while maintaining the same overall number of arginine residues, it is unlikely that a different clustering of R$_4$ and R$_8$ triggered the qualitative differences in the PMFs. Therefore, we speculated that the charged termini of the peptides might counteract the pore-stabilizing effects of the guanidium moieties, while termini play a larger role for the shorter R$_n$ owing to the increased number of termini *per* arginine. To test this hypothesis, we computed the PMF for tetra-arginine with electroneutral termini, denoted R$_4^\mathrm {cap}$. Evidently, replacing the charged with neutral termini stabilizes the pore by $\sim$12 kJ/mol; however R$_4^\mathrm {cap}$ remains less membrane-active than R$_8$ (Fig. 6b, dark green, blue, yellow curves). These results confirm that charged termini counteract the pore-stabilizing effects of arginine, rationalizing partly why only longer R$_n$ are membrane-active.

#### Thinning of the unperturbed membrane by R-derivatives only partly explains reduced $\varDelta G_\mathrm {pore}$ values

**Fig. 7:**
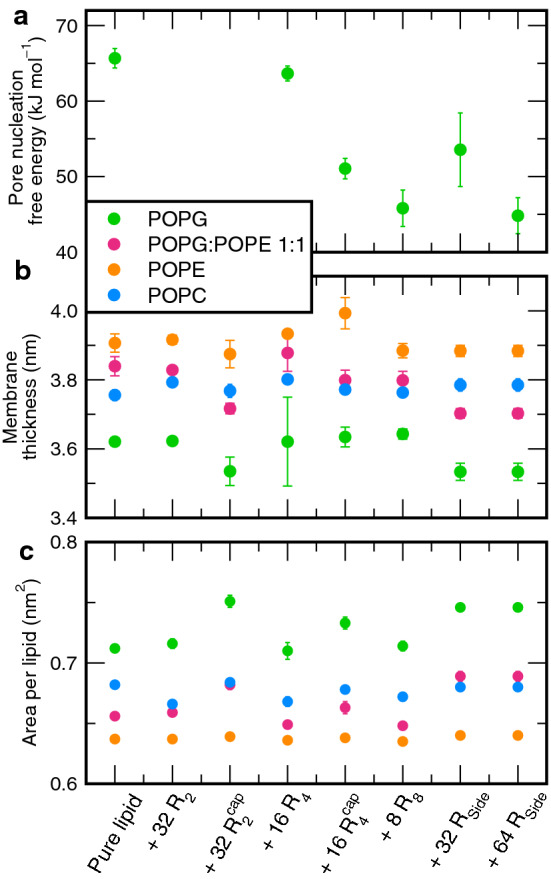
Modulation of membrane properties by the addition of polyarginines, derived from MD simulations: **a** free energy of pore nucleation, **b** membrane thickness, and **c** area per lipid. The lipid is encoded by color

It is well established that the free energy of pore formation correlates with membrane thickness (Bennett et al. 2014; Ting et al. 2018). To test if thinning of the flat, unperturbed membrane explains the effects of R-derivatives on $\varDelta G_\mathrm {pore}$, we computed the membrane thickness, defined as the mean distance between the phosphate atoms of the opposite leaflets (Fig. 7b), as well as the area per lipid (Fig. 7c). For reference, Fig. 7a summarized $\varDelta G_\mathrm {pore}$ values for POPG membranes. Here, destabilization of the membrane is indicated by a reduced thickness and increased area per lipid. Notably, membrane thickness anticorrelates with the area per lipid because the membranes are approximately incompressible. Evidently, the membrane-active R-derivatives R$_\mathrm {Side}$ and R$_4^\mathrm {cap}$, as well as R$_2^\mathrm {cap}$ lead to an increased area per lipid and (with the exception of R$_4^\mathrm {cap}$) to a reduced membrane thickness, indicating slightly more disordered tails. In contrast, the highly membrane active R$_8$ has no effect on membrane structure. Furthermore, the membrane thickness is reduced by less than 0.1 nm; in the light of previous studies that modulated the membrane thickness by simulating lipid with different tail lengths, this slight reduction of membrane thickness should lead to a stabilization of the pore by only a few kilojoules per mole (Bennett et al. 2014; Ting et al. 2018).

Hence, the effects of R-derivatives on membrane structure mainly reflect interactions of R-derivatives with the membrane, but the structural properties do not quantitatively explain their pore-stabilizing effects. Along these lines, the fact that structural parameters of zwitterionic POPE and POPC membranes are much less affected by R-derivatives reflects weak arginine–membrane interactions in those membranes. In turn, this analysis suggests that direct interaction of R-derivatives with the defect play important roles in pore formation.

#### R$_8$ stabilized a pore by connecting a transmembrane water defect with the headgroup region

**Fig. 8:**
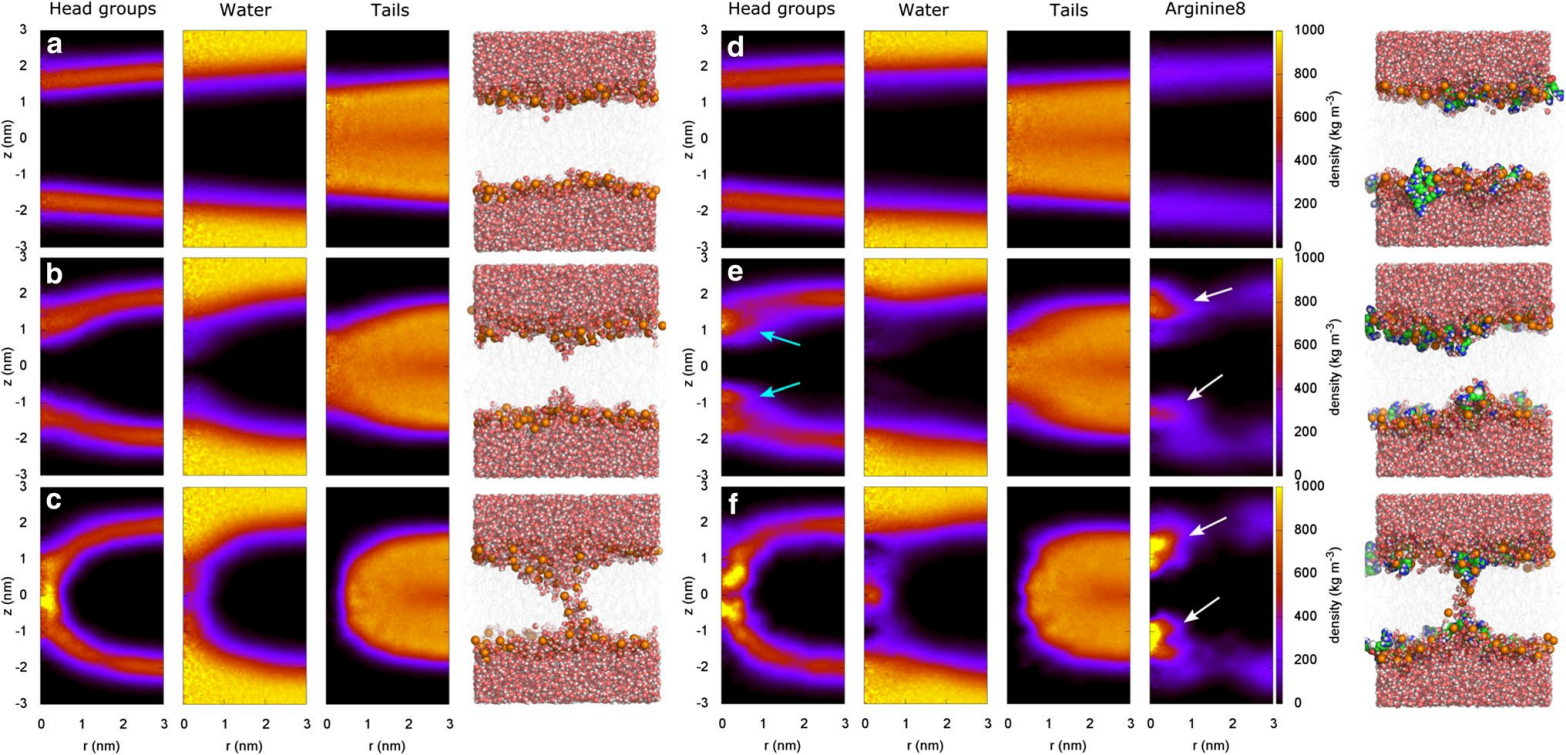
Pore formation during MD simulations over membranes of (**a**–**c**) pure POPG and (**d**–**f**) of POPG plus eight R$_8$. The columns in shaded colors show the mass density of (from left to right) headgroups, water, tails, and R$_8$, as averaged over umbrella sampling windows restrained at (A/D) $\xi _\mathrm {ch} = 0.225$, (B/E) $\xi _\mathrm {ch} = 0.625$, and $\xi _\mathrm {ch} = 1$, corresponding states with a flat membrane, a partial defect, and a fully formed pore, respectively. Densities are plotted as function of lateral distance *r* and vertical distance *z* from the center of the defect. Evidently, R$_8$ accumulates at the partial and fully formed defect (white arrows), where R$_8$ replaces water and stabilizes lipid headgroups (cyan arrows)

Figure 8 presents structural insight into the mechanism by which R$_8$ stabilized a pore by comparing pore formation over a POPG membrane in absence (Fig. 8a–c) and presence of R$_8$ (Fig. 8d–f). The colors represent the mass densities of headgroups, water, tails, and arginine (if present), as indicated on top of the columns, averaged over the respective umbrella windows at three stages of pore formation: flat membrane (top row, A/D), local thinning of the membrane corresponding to a partial defect characterized by an indentation of the headgroups (middle row, B/E), and a fully formed defect (bottom row, C/F).

We find that R$_8$ accumulates both at the partial and at the fully formed defect (Fig. 8e, f, white arrows), confirming that direct R$_8$-defect interactions play an important role. R$_8$ accumulates at the glycerol region of the membrane, where R$_8$ (1) stabilizes lipid headgroups that partially penetrate the membrane core (Fig. 8e, cyan arrows) and (2) where R$_8$ replaces water (Fig. 8e, f, second column, compare with B/C, second column). Consequently, in contrast to the pore over the pure-POPG membrane, the open pore in the R$_8$-containing membrane does not require a continuous transmembrane water file. Together with the PMFs, these data demonstrate that the accumulation of R$_8$ at the pore is energetically favorable and thereby facilitating pore formation.

## Discussion

Our data show that pore formation or membrane destabilization caused by arginine derivatives unfolds only in the presence of a high anionic lipid content. Concretely, only POPG-containing membranes are noticeably affected by arginine derivatives and in experiments pore formation by poly-arginine could only be achieved in pure POPG vesicles. This likely has consequences for pore formation and permeabilization to large molecules. The pore-forming mechanism introduced by Allolio et al. was experimentally supported by leakage assays in a PS/PE lipid mixture, while, in accordance with our data, POPC-containing membranes showed much less leakage (Allolio et al. 2018). Our findings combined with earlier studies imply that POPC has a protective effect against pore formation or membrane permeabilization by guanidine-rich molecules, further supported by our observation that poly-arginines seem to rather increase the line tension of a solid-supported POPC:POPG 1:1 bilayer.

The finding that leakage of membranes is only observed in membranes without PC-content indicates that besides being accepted as one of the most important amino acids for membrane-active peptides’ activity, arginine could also play a role in the apparent duality of some CPPs as AMPs and vice versa (Alvaro et al. 2016; Splith and Neundorf 2011; Henriques et al. 2006; Han and Patil 2000; Bahnsen et al. 2013; Budagavi and Chugh 2018), if one considers differences in lipid compositions between mammalian and bacterial membranes. A mammalian cell membrane typically contains at least 40% PC headgroups (van Meer et al. 2008). In contrast, bacterial membranes have a high content of PG or other anionic lipids and less PC (Shaw 1974; Sohlenkamp and Geiger 2016). Hence, mammalian cells might be protected by PC headgroups from formation of larger pores which could otherwise lead to cytotoxicity, while many bacterial membranes cannot rely on this protection from lysis. Instead, Gram-negative bacteria are likely protected by lipopolysaccharides covering the outer membrane (Berglund et al. 2015). It would be highly interesting to investigate the validity of this view, and to identify other protective components in mammalian cell membranes that might be absent in bacterial membranes. Recently for example, it was shown that several arginine-rich CPPs influence the cholesterol phase distribution in LUVs, with many favoring the liquid disordered phase (Almeida et al. 2019), but the influence of cholesterol on pore formation by arginine-rich compounds is not yet understood. Alternatively, factors in lipid composition that favor larger pore formation by arginine-rich peptides could also be investigated.

Several previous MD studies revealed pore formation by arginine-rich peptides, however these studies did not yield a consensus on the mechanism of the peptides at a quantitative or energetic level. As argued here, such spontaneous R$_n$ insertion in unbiased simulations is not needed to rationalize pore formation by R$_n$ because merely a *reduction* of the pore free energy in a small simulation patch is sufficient to rationalize the formation of (a few) pores in larger, experimentally relevant membranes. Hence, we believe that free energy calculations rather than unbiased simulations provide a route to a quantitative understanding of membrane-active peptides.

Based on PMF calculations, Sun et al. found only a small effect of R$_8$ on pore formation over a zwitterionic dipalmitoyl-*sn*-glycero-3-phosphocholine (DPPC) (Sun et al. 2014), in line with our findings that R$_8$ is only active in anionic membranes. Differences between the present study and the work by Sun et al. are further rationalized by the use of different reaction coordinates (RCs) for pore formation used during PMF calculations. First, Sun et al. used the distance of a DPPC headgroup from the membrane center as RC, corresponding to a half-way lipid flip-flop transition. However, this RC may lead to major hysteresis effects (Sun et al. 2014; Awasthi and Hub 2016), suggesting that the PMFs must be taken with care. The chain coordinate $\xi _\mathrm {ch}$ used here does not face such hysteresis problems (Hub and Awasthi 2017). Second, Sun et al. reported the PMF for translocating R$_8$ to the membrane center. During the formation of the polar defect studied here, in contrast, the penetration of R$_8$ into the membrane core is not required, as revealed by the R$_8$ densities at the open pore (Fig. 8). Instead, for opening the pore, R$_8$ merely accumulated below the headgroups, where they stabilized a polar defect of water and POPC headgroups (Fig. 8e, f, white arrow). Taken together, we conclude that R$_8$ acts predominantly by reducing the free energies of pore formation in anionic membranes, although kinetic effects discussed by Sun et al. may play a role in stabilizing the pore as well.

Further, continuum models (Ramírez et al. 2019) as well as simulations with the MARTINI coarse-grained force field were used to study membrane-active peptides. Hu et al. suggested that R$_8$ favors pore formation in asymmetric lipid bilayers, with 50% PS in their inner leaflet and the rest of the bilayer consisting of DMPC (Hu et al. 2019). However, the MARTINI coarse-grained force field models electrostatic interactions and hydrogen bonds only qualitatively, which likely play a critical role for arginine–membrane interactions. Hence, it will be interesting to test predictions from coarse-grained simulations with more quantitative simulations.

We found experimentally and in MD simulations that R$_{\text {Side}}$ and longer R-peptides display the highest membrane activity in anionic membranes. However, both approaches also indicate that the mechanisms of how the two arginine derivatives interact with anionic membranes differ. We observed that R$_{\text {Side}}$ rather partitions into the bilayer and thereby induces an overall destabilization, as evidenced by (1) membrane thinning and area per lipid expansion in MD simulations, (2) reduced $\varDelta G_\text {pore}$, and (3) the significant reduction of breakthrough forces in our experiments. Concretely, we found experimentally a decrease of both spreading pressure and line tension after administration of R$_{\text {Side}}$. Breakthrough forces in the presence of R$_{\text {Side}}$ decrease mainly due to a decrease line tension and spreading pressure that might be attributed to a increase in solubility of membrane patches by partitioning of R$_{\text {Side}}$. Successful leakage experiments with vesicles and dissolution of solid supported bilayers as observed by RIfS at higher concentrations further confirm this mode of action for R$_{\text {Side}}$.

In contrast, long polyarginines stabilize pores in MD simulations by direct interactions with the pore, as shown by decreased $\varDelta G_\mathrm {pore}$ (Fig. 6). Interestingly, polyarginines act without typical signs of overall bilayer destabilization, but instead by direct interactions with the polar defect (Fig. 8). Experimentally, longer $\text {R}_n$ are clearly membrane-active as revealed by the leakage assay in experiments (Fig. 4) and by the jump-to-contact observed in force curves, but they did not substantially reduce the breakthrough force in AFM experiments (Fig. 3). This apparent discrepancy might be explained by the presence of POPC in solid-supported membranes in AFM experiments: leakage could not significantly be achieved in POPC:POPG 1:1 vesicles. Another possibility is the larger pore radii generated by AFM indentation, as compared to the pore radii required for leakage or for forming a transmembrane defect. Accordingly, the increased line tension in the presence of R$_{12}$ might increase the free energy of the larger pores formed by the AFM tip, and thereby outweigh the stabilization of the small pore probed in simulations. Clearly, additional experiments and simulations will be required to fully resolve the role of $\text {R}_n$ during breakthrough experiments.

We further investigated the role of charged termini on free energies of pore formation in presence of the short polyarginine $\text {R}_4$. Remarkably, we found that $\text {R}_4$ with neutral termini is membrane-active, while $\text {R}_4$ with regular charged termini is not, suggesting that charged termini counteract the pore-stabilizing effects of arginine side chains. This finding may explain why short $\text {R}_n$ with a small number or arginine side chains per terminus are not membrane-active, even at high concentration.

## Conclusions

We have demonstrated experimentally and by simulations that both an arginine side chain mimic and longer poly-arginines facilitate pore formation in negatively charged model membranes, but that they do so via different mechanisms. The arginine side chain mimic R$_\mathrm {Side}$ causes overall destabilization of anionic membranes. This was evident from membrane thinning and lateral expansion in simulations, as well as from reduction of breakthrough forces and dissolution of membranes in experiments. However, longer R$_n$ did not show signs of overall destabilization, neither experimentally, nor in simulations, even though they did stabilize pores under specific conditions. This suggests a role of the peptide backbone in pore formation by poly-arginines, which is further supported by simulations showing that shorter oligo-arginines did not reduce the free energy associated with pore formation. Notably, the charged termini of shorter oligo-arginines counterbalance the pore-stabilizing effects of arginines, rationalizing why only longer poly-arginines are membrane-active.

Experiments further revealed that negative lipids were critical for pore formation and that PC headgroups seem to protect from pore formation by guanidine-rich molecules. Polyarginines caused a rise of line tension in membranes containing 50% PC headgroups and it was necessary to use pure POPG vesicles to provoke leakage of encapsulated dyes. We suggest that this observation may help to explain the regularly reported antimicrobial activity of arginine-rich CPPs.

## Supplementary Information

Below is the link to the electronic supplementary material.

Supplementary file1 (PDF 1737 KB)
