# Supplementary material for: How arginine derivatives alter the stability of lipid membranes: dissecting the roles of side chains, backbone and termini
Source: Eur Biophys J. 2021 Mar 4;50(2):127–42. doi: 10.1007/s00249-021-01503-x (PMC8071801; doi:10.1007/s00249-021-01503-x)
Supplement: Supplementary file 1 — Supplementary file1 (PDF 1737 KB) [file 249_2021_1503_MOESM1_ESM.pdf]

Eur. Biophys. J. manuscript No.
(will be inserted by the editor)

Supporting Information: How arginine derivatives alter the stability of lipid membranes: dissecting the roles of sidechains, backbone, and termini

Sarah F. Verbeek[1], Neha Awasthi[2], Nikolas K. Teiwes[1], Ingo Mey[1], Jochen S. Hub[a,2,3,c], Andreas Janshoff[b,1,c]

[1]Georg-August-Universität Göttingen, Department of Chemistry, Institute of Physical Chemistry, Göttingen 37077, Germany
[2]Georg-August-Universität Göttingen, Institute of Microbiology and Generics, Göttingen 37077, Germany
[3]Saarland University, Theoretical Physics and Center for Biophyics, Saarbrücken 66123, Germany

Received: date / Accepted: date

1 Methods

1.1 Leakage assays

2mg lipid films for leakage assays in the desired lipid composition were hydrated in 400 μL of desired buffer of measuring buffer (50 mM Na_2HPO_4, 0.1 mM EDTA) and left to swell as described in the main article. After suspension, they were extruded 31 times over a 100 nm polycarbonate filter (Avestin Europe) using an Avestin LiposoFast mini extruder system to yield LUVs. To exchange the buffer outside the vesicles for a buffer without dye, vesicles were added to an Illustra^{TM} NAP^{TM} disposable column prepacked with Sephadex^{TM} G-25 DNA grade (GE Healthcare) which was pre-equilibrated with 20 mL of measuring buffer. Subsequently, 2 mL of measuring buffer were added slowly. Next, 4 x 500 μL of measuring buffer were added, and these fractions were collected (always fraction 1-3, so 1.5 mL of vesicles in total of approximately 0.75 mg/mL lipid concentration). The fractions that visually contained most of the dye were combined and stored for a leakage assay. The procedure yielded a ca. 2.5× dilution of the vesicles, which was used to calculate the estimated peptide-to-lipid ratios.

Leakage assays with sulforhodamine B (ordered from Sigma) were performed on a Jasco-FP 6500 spectrofluorometer. Samples were prepared as 1 mL containing 1 μL of vesicle solution in 999 μL of measuring buffer in disposable 1.5 mL PMMA cuvettes (Brand). Sulforhodamine B fluorescence intensity was monitored over time at 565 nm excitation and 585 nm emission wavelengths. The solution was stirred continuously by a 3×3 mm micro stirring bar (VWR International). R_9 or R_{Side} was added after ca. 300 s at the desired concentration (from stock solutions that had 100x the desired concentration in measuring buffer, resulting in additions

[a]e-mail: jochen.hub@uni-saarland.de
[b]e-mail: ajansho@gwdg.de
[c]These authors jointly supervised the project.

of ca. 10 µL). 100% leakage was determined by addition 20 µL of 10-fold diluted Triton X-100 in measuring buffer. Sulforhodamine B was normalised to 0% leakage for its fluorescence intensity at the start of the assay and to 100% for its intensity after addition of Triton X-100 solution. Data were corrected for linear drift by subtracting a line fitted to the fluorescence data before R6 addition.

Peptide-to-lipid ratio could thus not be determined experimentally by phosphate test, however, the consistent appliance of experimental conditions took priority. Peptide-to-lipid ratios can be estimated to be 5.88:1 for 10 µM R_9 and 5882:1 for 100 mM R_{Side}.

1.2 Reflectometric interference spectroscopy

A similar procedure for RIfS was described before [1]. For reflectometric interference spectroscopy, P-type, boron-doped silicon wafers of 525 µm thickness with a 5000 nm SiO_2-layer and a resistivity of 5-10 Ωcm were used. They were cut to ca. 6 mm × 19.5 mm and cleaned in a basic piranha solution (5:1:1 v/v/v H_2O:25%NH_4OH:30% H_2O_2) at 70 °C for 20 minutes, thoroughly rinsed with water and stored in ultrapure water before use. Before use, they were thoroughly rinsed with water and ethanol p.a. and dried under N_2 stream. Subsequently, they were treated in a Diener Zepto plasma cleaner as follows: 5.5 minutes of oxygen flow at 0.20 mbar, followed by a 30s plasma process at 60% power. Wafers were inserted into custom made flow cells consisting of an aluminium base with an acrylic glass cover similar to those described by Stephan *et al.* [2] except that the aluminium base was flat instead of indented.

Reflectometric interference spectroscopy was performed using a tungsten halogen light source (HL-2000-FHSA Ocean Optics) combined with a Ocean Optics flame miniature spectrometer (FLAME-S-UV-VIS-ES, Ocean Optics). A reflection spectrum between 500 and 700 nm wavelengths was recorded every 2 s using SpectraSuite software (Ocean Optics). Polished aluminium was used as the reference for full reflection of the illuminating light. Calculation of optical thickness OT was performed live during the experiment using a Matlab-based graphical user interface. Flow rate in all experiments was 0.43 mL min^{-1}. All buffers were newly degassed before a RIfS experiment. The system was hydrated in ultrapure water, which was exchanged for spreading buffer (20 mM trisodium citrate, 50 mM KCl, 0.1 mM EDTA, 0.1 mM NaN_3, pH 4.8) when the system was stable. To form the solid-supported lipid bilayer, 200 µL of vesicle solution was added to the system POPC:POPG:BP 49.5:49.5:1 prepared as described in main article), and it was left to circulate as a closed system for 45 minutes. For determination of dissociation constants of R-peptides, vesicles were rinsed out of the system by a 20 minutes flow in an open system with measuring buffer. Then, R-peptide was added in increasing amounts in a closed system fashion. To obtain the dissociation constant for a peptide, combined data from at least 2 experiments were fitted to an adapted Langmuir equation:

$$\Delta OT = \frac{\Delta OT_{max} \times [\mathrm{R_n}]}{K_D + [\mathrm{R_n}]} \tag{1}$$

Where ΔOT is the optical thickness change with respect to the optical thickness of the membrane before addition of R-peptide, ΔOT_{max} is the maximal op-

tical thickness change reached, $[\mathrm{R_n}]$ is the peptide concentration, and K_D is the dissociation constant.

2 Supplementary tables and figures

Table 1 Dissociation constants for R-peptides of different lengths as determined on POPC:POPG:BP 49.5:49.5:1 bilayers by RIfS, illustrating that the concentrations used in AFM experiments should ensure sufficient coverage of the surface being the same order of magnitude as the dissociation constants.

	$\mathrm{R_2}$	$\mathrm{R_6}$	$\mathrm{R_8}$	$\mathrm{R_{12}}$
K_D (µM)	$(22 \pm 6.0) \times 10^{-3}$	7 ± 1.5	1.5 ± 0.39	0.7 ± 0.14

Table 2 On the influence of using an integration time step of $\Delta t = 4\,\mathrm{fs}$ and of constraining the C-O-H angles of the POPG hydroxyl groups on the structure of the POPG membrane: area per lipid A_L, number of lipid-lipid hydrogen bonds N^h_LL, and number of hydrogen bonds between water and the hydroxyl groups of DOPG $N^\mathrm{h}_\mathrm{OH\text{-}W}$ per lipid. The properties were computed from 200 ns simulations of 128 POPG lipids plus 6221 water molecules, omitting the first 30 ns for equilibration. Evidently, constraining the C-O-H angle and using a 4 fs time step has only a marginal effect on these membrane properties. Errors were computed by block averaging with the Gromacs module gmx analyze.

	A_L (Å^2)	N^h_LL	$N^\mathrm{h}_\mathrm{OH\text{-}W}$
no angle restraint, $\Delta t = 2\,\mathrm{fs}$	70.06 ± 0.18	2.24 ± 0.01	4.09 ± 0.02
COH angle restraint, $\Delta t = 2\,\mathrm{fs}$	70.05 ± 0.35	2.21 ± 0.01	4.13 ± 0.03
COH angle restraint, $\Delta t = 4\,\mathrm{fs}$	70.15 ± 0.35	2.20 ± 0.01	4.16 ± 0.02

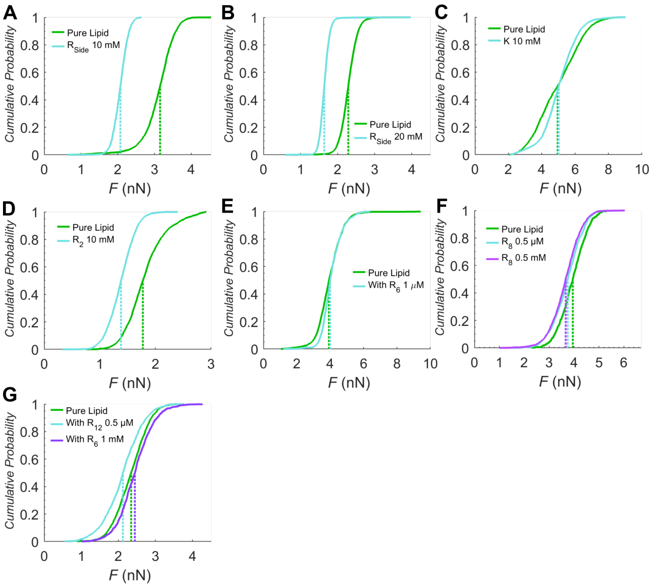

Fig. 1 Representative yield force data for POPC:POPG:BP 49.5:49.5:1 bilayers, to illustrate that the yield force F was between 1-6 nN depending on cantilever used. Each plot represents data recorded within one experiment and with the same cantilever. First, data were recorded on the membrane without R-peptide or R_{Side}, then R-peptide was added to the desired concentration. At least 2 experiments (usually 3) were performed per combination of lipid mixture and peptide, and only one experiment per R-peptide is shown here. (A) R_{Side} 10 mM; (B) R_{Side} 20 mM; (C) Lysine 10 mM; (D) R_2 10 mM; (E) R_6 1 μM (F) R_8 (G) R_{12} 0.5 μM and R_6 1 mM.

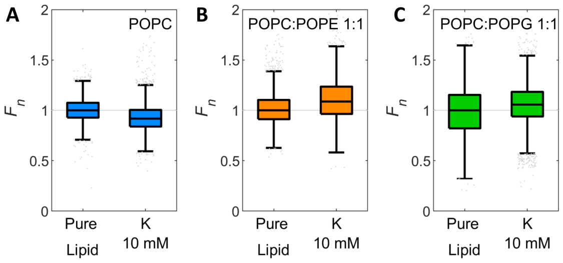

Fig. 2 Normalized yield force results F_n for all employed lipid mixtures with addition of lysine (K) as a control experiment. Normalization was performed for the median of the yield force without peptide for each experiment. Each boxplot contains combined data from at least two experiments.The bottom and top edge of the boxplots indicate the 25th and 75th percentile of each data set, and the line dividing the box indicates the median. The upper and lower whisker represent approximately 2.7 standard deviations higher or lower than the mean, respectively. Outliers (points beyond 2.7 standard deviations from the mean) are shown as grey points. (A) Results for POPC; (B) Results for POPC:POPE 1:1; (C) Results for POPC:POPG 1:1.

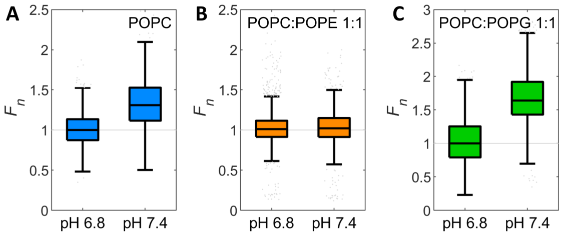

Fig. 3 Normalized yield force results F_n for all employed lipid mixtures under a buffer exchange from pH 6.8 (used with R-peptides as well) to pH 7.4, to investigate whether the observed effects for R_{Side} are purely explained to a pH rise due to its basicity. Normalization was performed for the median of the yield force without peptide for each experiment. Each boxplot contains combined data from at least two experiments.The bottom and top edge of the boxplots indicate the 25th and 75th percentile of each data set, and the line dividing the box indicates the median. The upper and lower whisker represent approximately 2.7 standard deviations higher or lower than the mean, respectively. Outliers (points beyond 2.7 standard deviations from the mean) are shown as grey points. (A) Results for POPC; (B) Results for POPC:POPE 1:1; (C) Results for POPC:POPG 1:1.

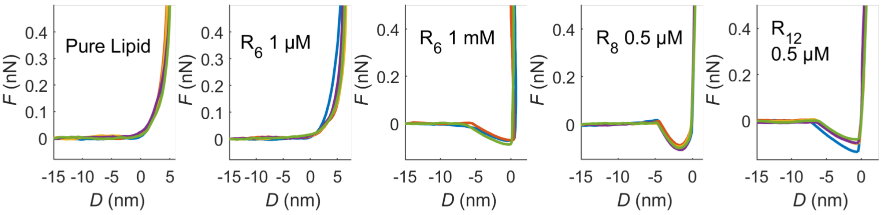

Fig. 4 Example approach force curves zoomed in close to the contact point, for POPC:POPG 1:1 as compared to its combination with several oligo-arginines in different concentrations; The increasingly pronounced jump-to-contact (brief negative force upon contact) for longer peptides in lower concentrations illustrates the influence of R-peptides on the interaction between the cantilever tip and the bilayer.

References

1. R. Krick, R.A. Busse, A. Scacioc, M. Stephan, A. Janshoff, M. Thumm, K. Kühnel, PNAS **109**(30), E2042 (2012). DOI 10.1073/pnas.1205128109. URL https://www.pnas.org/content/109/30/E2042. Publisher: National Academy of Sciences Section: PNAS Plus
2. M. Stephan, C. Kramer, C. Steinem, A. Janshoff, Analyst **139**(8), 1987 (2014). DOI 10.1039/C4AN00009A. URL https://pubs.rsc.org/en/content/articlelanding/2014/an/c4an00009a. Publisher: The Royal Society of Chemistry
